# Dissociation of understanding from applying others’ false beliefs in remitted schizophrenia: evidence from a computerized referential communication task

**DOI:** 10.1186/1471-244X-13-141

**Published:** 2013-05-17

**Authors:** Yong-guang Wang, David L Roberts, Bai-hua Xu

**Affiliations:** 1 Department of Psychology and Behavioral Sciences, Zhejiang University, 148 Tianmushan Road, Hangzhou, Zhejiang Province 310028, China; 2 Department of Mental Health, Hangzhou Center for Disease Control and Prevention, Hangzhou, Zhejiang Province, China; 3 Department of Psychiatry, University of Texas Health Science Center, San Antonio, USA

**Keywords:** Theory of mind, Schizophrenia, False belief, Referential communication task, Perspective taking

## Abstract

**Background:**

In research on theory of mind (ToM), false belief paradigms are commonly used. Previous studies have reported that there is heterogeneity in the magnitude of impairment on false belief tasks. Moreover, intact ability to attribute others’ false beliefs has been widely reported in patients with remitted schizophrenia. Increasingly, evidence suggests that there may be different cognitive mechanisms underlying the understanding others’ false beliefs versus applying one’s knowledge of others’ false beliefs. Since the role of psychotic symptoms in ToM impairments is an important issue in the study of ToM deficits in schizophrenia, we examined both remitted schizophrenia and non-remitted schizophrenia, with the aim to investigate whether psychotic symptoms in schizophrenia are associated with deficits in understanding others’ mental states or difficulties in applying this understanding.

**Methods:**

The present study investigated 29 patients with non-remitted schizophrenia, 19 patients with remitted schizophrenia, and 22 healthy controls with a revised computerized referential communication task. The ability to understand others’ false beliefs and the ability to apply others’ false beliefs were measured separately.

**Results:**

Patients with non-remitted schizophrenia performed significantly worse than patients with remitted schizophrenia and healthy controls on a task of understanding others’ false beliefs, whereas no significant difference was found between the patients with remitted schizophrenia and healthy controls. Both the patients with non-remitted schizophrenia and patients with remitted schizophrenia performed significantly worse than healthy controls on a task of applying others’ false beliefs.

**Conclusions:**

Our findings suggested a dissociation of understanding others’ false beliefs from applying others’ false beliefs in remitted schizophrenia. We preliminarily conclude that deficits in the ToM ability of applying knowledge of others’ mental states might be state-dependent.

## Background

Theory of mind (ToM) refers to the ability to understand others’ mental states, such as beliefs, and intentions [1]. In the ToM field, false belief paradigms are commonly used. In a typical false belief task, an agent is depicted as having a belief that is inconsistent with reality (e.g., believing that a ball is in a basket when it is actually in a box), leading to a behavior that is incompatible with reality (e.g., looking for the ball in the basket). This task is used to investigate whether participants can predict an agent’s behavior based on the agent’s belief, despite knowing that the belief is inconsistent with reality. Studies in children using an elicited-response false belief task (e.g., the Sally-Anne false belief task) [2] suggest that four-year-old children can predict others’ behavior based on others’ false beliefs, whereas three-year-old children typically cannot [3-5]. However, developmental theorists using spontaneous-response false belief tasks have also documented that the ability to attribute others’ false beliefs is already present by the second year of life [2,6,7]. In these tasks, children’s understanding of others’ false beliefs is inferred from their spontaneous behaviors while they watch a scene unfold. For example, Onishi and Baillargeon [6] found that 15-month-old infants looked reliably longer when the agent searched for an object in one of two boxes when the agent falsely believed it to be in other box, suggesting that 15-month-old infants have the ability to understand others’ false beliefs. One explanation for these findings, the “response account,” holds that younger children fail the elicited-response false belief task because of difficulties in executing the response-selection and response-inhibition processes, but not in executing the false-belief-representation process [2].

Failure or processing costs in executing false belief tasks have also been reported in normal adults. Keysar et al. reported that normal adults did not reliably use others’ false beliefs to interpret their behavior in a referential communication task [8]. Other studies replicated the findings from Keysar et al. [8], and further suggested that applying knowledge of others’ false beliefs appears to require effort to complete [9,10]. On the contrary, several studies have suggested that the false-belief representation process might be automatic or spontaneous in normal adults. For example, Kovács et al. [11] found that participants’ reaction times in a visual object detection task were modulated by the false beliefs of a fictional agent even when participants were aware that the agent’s beliefs were unrelated to the task, suggesting that participants computed the agent’s beliefs automatically. Similarly, Cohen and German found that adults’ tendency to parse events involving a human agent in terms of the agent’s belief was unintentional [12,13]. Back and Apperly also found that belief ascription may occur spontaneously [14]. Considering studies from normal children and adults, it seems that there may be different cognitive mechanisms underlying the understanding others’ false beliefs versus applying knowledge of others’ false beliefs.

Since Frith proposed that psychotic symptoms in schizophrenia might be explained by ToM impairments [15], numerous studies have examined ToM in schizophrenia. While there is no doubt that schizophrenia patients perform poorly on ToM tasks, these studies have produced some variable findings. For example, studies have reported that there is heterogeneity in the magnitude of impairment on false belief tasks [16,17]. Moreover, inconsistent findings on false belief tasks have been widely reported in patients with remitted schizophrenia. For instance, Corcoran et al. [18] found that patients who were symptom free showed normal performance on a task of understanding others’ false beliefs from visual jokes. Other studies have also reported intact ability to understand others’ false beliefs in patients with remitted schizophrenia [19-22], suggesting that ToM deficits in schizophrenia are state dependent. In contrast, other studies have found that subjects with remitted schizophrenia performed poorly on the false belief picture-sequencing task [23,24]. For example, Inoue et al. [23] reported the patients in remission after the first episode of schizophrenia showed impairment on the false belief picture sequencing task, suggesting ToM deficits as a trait characteristic of schizophrenia.

In light of the findings among normal children and adults on false belief tasks, we speculate that differences in the nature of the tasks used to assess ToM might confound these results. Succeeding in false belief picture-sequencing tasks requires participants to rearrange the cards according the character’s false belief, which involves not only understanding others’ false beliefs but also the ability to apply this knowledge of others’ false beliefs. Considering studies from remitted schizophrenia, we hypothesized that patients with remitted schizophrenia may be impaired in applying their knowledge of others’ false beliefs but have intact ability to understand others’ false beliefs. Our hypothesis is consistent with previous findings [25,26]. Champagne-Lavau et al. [25] found that schizophrenia patients had problems using information they shared with the experimenter in a referential communication task. In particular, McCabe et al. [26] reported that the patients with chronic schizophrenia retained erroneous beliefs despite recognizing that their interlocutor did not share their belief or accept their justifications of these beliefs as convincing. These findings suggest that the problems facing these patients are not understanding others’ beliefs, but application of the knowledge of others’ mental states.

Thus, we conducted the present study to explore ToM ability in schizophrenia using a decompositional approach. A revised computerized referential communication task was adopted to investigate separately the ability to understand another’s false beliefs and the ability to apply understanding of the other’s false beliefs. Since the role of psychotic symptoms in ToM impairments is an important issue in the study of ToM deficits in schizophrenia [15], we examined both remitted schizophrenia and non-remitted schizophrenia, with the aim to investigate whether psychotic symptoms in schizophrenia are associated with deficits in understanding others’ mental states or difficulties in applying this understanding.

## Method

### Participants

Forty-eight patients with schizophrenia were recruited from the Seventh Hospital of Hangzhou (the largest psychiatric hospital of Zhejiang province) and the local community health institutes in the city of Hangzhou. The diagnosis of schizophrenia was determined by two experienced psychiatrists (YW and YM) according to the criteria of the Diagnostic and Statistical Manual of Mental Disorders 4th ED. [27]. Patients who had a current or past diagnosis of substance dependence, a severe medical or neurological condition, or other clinical pathologies that could be associated with poor social functioning were excluded. The patients were divided into a schizophrenia-non-remitted group (n=29) and a schizophrenia-remitted group (n=19) according to Andreasen’s remission criteria [28]. To serve as a control group, twenty-two normal healthy adults with no psychiatric history were recruited from the local community. All healthy adults met the following inclusion criteria: (a) no history of head injury, CNS disease or psychiatric illness, and (b) no evidence of current substance (including alcohol) abuse. All participants were over 18 years of age. They all had normal vision and hearing. All participants were right-handed.

**Figure 1:**
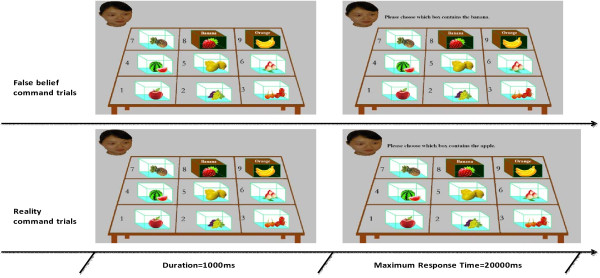
**Examples of false belief command trials and reality command trials.** Fist, a photograph was presented. The photograph was continued and a statement was presented until a response was made. In each photograph, the character named ZhangHong can see the fruits in the non-secretive boxes (No. 1 to 7) and the labels on the secretive boxes (No. 8 and No. 9), but not the fruit inside the secretive boxes. ZhangHong has false belief for the content of box No. 8 and box No. 9.

All participants in this study gave written, informed consent. Consent was obtained from the person in Figure 1 to publish the image in Figure 1.

### Measurements

#### Revised computerized referential communication task

A revised computerized referential communication task was used to investigate the ability to understand others’ false beliefs and to apply this understanding [10]. During each trial of the computerized referential communication task, a photograph was shown (Figure 1 left). Each photograph contains nine boxes (seven non-secretive boxes and two secretive boxes) with a different fruit in each box. Above each of the two secretive boxes is written a label supposedly indicating the name of the fruit in the box. In the top-left of the photograph is a picture of a fictional character, ZhangHong. The label above each secretive box is inconsistent with the content of the box. Participants were told that ZhangHong cannot see the content of secretive boxes, but can see the labels above the boxes as well as the content of the non-secretive boxes, which results in ZhangHong having false beliefs about the contents of the two secretive boxes. In addition, one of the secretive boxes is a critical secretive box for which the label corresponds to the content of the other secretive box (e.g., In Figure 1, the label of the No. 8 box corresponds to the contents of the No. 9 box).

### Applying others’ false beliefs

In the task of applying others’ false beliefs, a statement was presented at the top of the photograph (Figure 1 right), and participants were instructed that this was a command given to ZhangHong. Participants were instructed to take ZhangHong’s perspective and to predict her behavior. Nine “false belief command trials” and 18 “reality command trials” were presented. In the false belief command trials, the command statement referred to the fruit that is named on the critical secretive box but which is actually inside the other secretive box (e.g., “Please choose which box contains the banana.” Figure 1. top-right). If participants did apply their knowledge of the ZhangHong’s mental states, they would choose the critical secretive box as the box that ZhangHong would select (e.g., ZhangHong would incorrectly select the No. 8 box in Figure 1 in response to the command, “Please choose which box contains the banana.”). In the reality command trials, the statement made to ZhangHong referred to one of the other seven boxes (e.g., “Please choose which box contains the apple.” Figure 1 bottom-right), in which her belief corresponds with reality. To respond correctly in reality command trials, participants only needed to choose the target box according to their own knowledge of the contents of the seven non-secretive boxes. False belief command trials and reality command trials were presented in a pseudorandom order, avoiding consecutive false belief command trials in order to reduce the likelihood of participants inferring the purpose of the experiment. Participants were required to make each choice using the numbers on a numerical keyboard, and to be as accurate and fast as possible.

### Understanding others’ false beliefs

In the task of understanding others’ false beliefs, the experimental features were identical to those used in the task of applying others’ false beliefs with the exception of the command statements and response type. Two types of statements were used: false belief probes in which participants were asked to judge the accuracy of ZhangHong’s belief about the contents of specific boxes (e.g., “ZhangHong thinks that a banana is in box 8.”) and reality probes in which participants were asked to judge the truth about the contents of specific boxes (e.g., “In fact, the apple is in box 1.”). Participants were explicitly instructed that the probe statements are about either the truth of what is in the box or ZhangHong’s belief about what in the box. The participants were required to provide a “yes” or “no” button-press response to judge the accuracy of each probe sentence, and to be as accurate and fast as possible. A total of 9 false belief probes about the character’s false beliefs were presented in the trials corresponding to the false belief command trials in the task of applying others’ false beliefs. A total of 18 reality probes about the reality information were presented in the trials corresponding to the reality command trials in the task of applying others’ false beliefs. For false belief probes, 4 correct answers were “yes” and 5 correct answers were “no”. For reality probes, the answers were equally “yes” and “no”.

### Task procedure

The task procedure began with a name familiarization task for the ten kinds of fruits used in the experiment followed by the task of applying others’ false beliefs. Finally, all participants were administered the task of understanding others’ false beliefs. Before the formal experimental trials of the task of applying or understanding others’ false beliefs, four practice reality command/probe trials were administered to familiarize the participants with the task. The experiment was presented with DMDX software. Only the rate of accuracy for each condition was entered into the final analyses.

### Clinical evaluation

Information on sex, age, years of education, Chlorpromazine (CPZ) equivalents and length of illness was confirmed by psychiatrists and study researchers. Psychopathology was assessed using the Positive and Negative Syndrome Scale (PANSS) [29] for patients by experienced clinicians. Social functioning was evaluated using the Chinese version of the Personal and Social Performance Scale (PSP) [30] for patients by psychiatrists who have received training on the use of the PSP.

### Neuropsychological background tests and IQ Test

Since previous studies have reported that ToM deficits in schizophrenia were associated with impairments on IQ, executive functioning, and working memory [16,17,31], neuropsychological (i.e., the Verbal Fluency Test and The Digit Span Test) and IQ tests were administered to patients*.* The Verbal Fluency Test (VFT) was used to assess executive functioning and required patients to name vegetables within 1 min. The total score the patient could receive on the VFT was the number of vegetables named. The Digit Span Test (DST) was used to assess working memory including the Digit Span Forward Test and the Digit Span Backward Test. In the Digit Span Test, a series of lists of numbers is presented verbally to participants. The participant is asked to recall the numbers in ascending numerical order (forward) or descending numerical order (backward). The total score is the number of lists that are correctly remembered in ascending numerical order and descending numerical order. The Wechsler Adult Intelligence Scale-Revised Chinese Version (WAIS-RC) [32] was administered as a measure of intelligence for patients.

## Results

### Demographic, clinical data, and performance on neuropsychological tests

Table 1 summarizes the demographic characteristics, clinical data, and performance on neuropsychological tests for each group. Pearson’s Chi-square test was carried out to assess the Sex Ratio among groups. Parametric one-way analyses variance (ANOVAs) were performed for Index age, Educational level, Length of illness, CPZ equivalents, PSP total score, PANSS General symptoms, PANSS Positive symptoms, PANSS Negative symptoms, VFT total score, DST total score and IQ. The levels of statistical significance were set at least at *P* < 0.05.

**Table 1:** Comparisons of demographic data, clinical data, and performance on neuropsychological tests among groups

	**Healthy controls**	**Remitted schizophrenia**	**Non-remitted schizophrenia**	**Statistics**
	**(n=22)**	**(n=19)**	**(n=29)**	
Sex ratio (M: F)	9:13	8:11	13:16	*χ*^*2*^=0.084, *P*=0.959
Index age (years)	31.86±5.36	34.89±5.26	31.86±12.73	*F*(2,138)=0.756, *P*=0.473
Educational level (years)	11.05±1.73	10.47±2.37	11.52±2.59	*F*(2,138)=1.198, *P*=0.308
Length of illness (years)		11.68±6.06	8.17±9.64	*F*(1,47)=1.996, *P*=0.164
CPZ equivalents (mg)		326.84±157.13	309.48±164.40	*F*(1,47)=0.132, *P*=0.718
PSP total score		65.37±7.31	57.07±17.12	*F*(1,47)=3.966, *P*=0.052
PANSS				
General symptoms		24.68±3.40	31.17±5.56	*F*(1,47)=20.693, *P*<0.001
Positive symptoms		13.11±2.60	16.41±4.24	*F*(1,47)=9.251, *P*=0.004
Negative symptoms		16.11±2.28	18.21±4.21	*F*(1,47)=3.949, *P*=0.053
VFT total score		16.89±4.85	16.41±5.00	*F*(1,47)=0.109, *P*=0.743
DST total score		11.11±1.91	11.55±2.54	*F*(1,47)=0.426, *P*=0.517
IQ		96.32±11.60	97.90±10.35	*F*(1,47)=0.243, *P*=0.624

The non-remitted schizophrenia group had higher scores than remitted schizophrenia group on the PANSS General symptoms scales [*F*(1,47)=20.693, *P*<0.001] and PANSS Positive symptom scales [*F*(1,47)=9.251, *P*=0.004]. The differences between the non-remitted and remitted schizophrenia groups on the PSP [*F*(1,47)=3.966, *P*=0.052] and PANSS Negative symptoms scales [*F*(1,47)=3.949, *P*=0.053] approached statistical significance. No other significant differences were found between the remitted and non-remitted schizophrenia groups or among the three groups (all *Ps*>0.16).

### Performance on the revised computerized referential communication task

Table 2 summarizes the rates of accuracy for each condition on the revised computerized referential communication task among groups. A multiple one-way ANOVA was first conducted for rate of accuracy among groups. Post-hoc Bonferroni corrections were used to correct for multiple comparisons. The levels of statistical significance were set at least at *P* < 0.05.

**Table 2:** Comparisons of rate of accuracy for each condition on the revised computerized referential communication task among groups

	**Healthy controls**	**Remitted schizophrenia**	**Non-remitted schizophrenia**	**Statistics**
	**(n=22)**	**(n=19)**	**(n=29)**	
Task of applying				
False belief command	0.87±0.13	0.32±0.40	0.27±0.41	*F*(2,138)=22.204, *P*<0.001
Reality command	0.99±0.02	0.99±0.03	0.98±0.03	*F*(2,138)=1.230, *P*=0.299
Task of understanding				
False belief probe	0.97±0.05	0.87±0.25	0.53±0.39	*F*(2,138)=16.586, *P*<0.001
Reality probe	0.99±0.02	0.98±0.03	0.97±0.04	*F*(2,138)=1.441, *P*=0.244

For the task of applying understanding of false beliefs, a significant main effect for group was found on belief command trials [*F*(2,138)=22.204, *P*<0.001], but not for reality command trials [*F*(2,138)=1.230, *P*=0.299]. Post-hoc comparisons (Bonferroni corrected) showed that both the remitted schizophrenia group (*P*<0.001) and the non-remitted schizophrenia group (*P*<0.001) performed worse than healthy controls on false belief command trials. No other post-hoc comparisons were statistically significant (all *Ps*>0.364). For the task of understanding false beliefs, a significant main effect for group was found on false belief probe trials [*F*(2,138)=16.586, *P*<0.001], but not on reality probe trials [*F*(2,138)=1.441, *P*=0.244]. Post-hoc comparisons (Bonferroni corrected) showed that the non-remitted schizophrenia group performed worse than both the remitted schizophrenia group (*P*=0.001) and healthy controls (*P*<0.001) on false belief probe trials. No other significant differences between groups were found during post-hoc comparisons (all *Ps*>0.302). To examine further the significant difference between the non-remitted and remitted schizophrenia groups on the task of applying false beliefs, we added the rate of accuracy of false belief probe trials as a covariate into the an ANCOVA to exclude the possibility that failure in applying false beliefs was due to lack of understanding of others’ false beliefs. A significant difference was still found between the non-remitted and remitted schizophrenia groups on the task of applying false beliefs [*F*(1,47)=2.84, *P*=0.027].

### Correlations between performance on false belief trials, neurocognitive domain scores and clinical variables in schizophrenia

Since previous research has documented that IQ [16,17], antipsychotic treatment [33], length of illness [34], working memory [16,17,30], and executive functioning [16,17] are associated with ToM deficits in schizophrenia, Pearson’s correlations were conducted between performance on false belief trials, neurocognitive domain scores and clinical variables in schizophrenia participants. These variables included the rate of accuracy on trials of false belief probe, rate of accuracy on trials of false belief command, PSP total score, PANSS General symptoms, PANSS Positive symptoms, PANSS Negative symptoms, IQ, CPZ equivalents, Length of illness, VFT total score, and DST total score. Since a large number of correlations were examined in this analysis, the levels of statistical significance were set at least at *P* < 0.01 to limit the possibility of a Type I error. All correlations are shown in Table 3.

**Table 3:** Correlations between performance on false belief trials, neurocognitive domain scores and clinical variables in schizophrenia

	**False belief command**	**PANSS positive symptoms**	**PANSS negative symptoms**	**PANSS general symptoms**	**PSP total score**	**IQ**	**CPZ equivalents**	**Length of illness**	**VFT total score**	**DST total score**
False Belief Probe	0.598^*^	−0.066	−0.066	−0.368^**^	0.116	0.107	0.268	−0.129	−0.010	−0.133
False Belief Command		0.197	−0.035	−0.084	−0.062	0.012	0.165	−0.192	0.223	−0.018
PANSS Positive symptoms			0.117	0.249	−0.599^*^	−0.057	−0.169	−0.169	0.180	0.070
PANSS Negative symptoms				0.447^*^	−0.302	0.129	−0.339	−0.197	−0.325	−0.044
PANSS General symptoms					−0.397^*^	−0.104	−0.186	−0.090	−0.142	−0.103
PSP total Score						0.036	0.210	0.169	−0.013	0.107
IQ							0.094	−0.387^*^	0.242	0.243
CPZ equivalents								−0.075	0.244	0.022
Length of illness									−0.304	−0.211
VFT total score										0.425^*^

^*^P< 0.01; ^**^P= 0.01.

The correlation between rate of accuracy on trials of false belief probe and PANSS General symptoms (*r*=−0.368, *P*=0.010) approached statistical significance. There was a significant positive correlation between rate of accuracy on trials of false belief probe and accuracy on trials of false belief command (*r*=0.598, *P*<0.001), a significant positive correlation between PANSS General symptoms and PANSS Negative symptoms (*r*=0.447, *P*=0.001), and a significant negative correlation between IQ and Length of illness (*r*=0.387, *P*=0.007). There was a significant negative correlation between PSP total score and PANSS General symptoms (*r*=−0.397, *P*=0.005), and a significant negative correlation between PSP total score and PANSS Positive symptoms (*r*=−0.599, *P*<0.001). There was a significant positive correlation between DST total score and VFT total score (*r*=0.425, *P*=0.003). No other significant correlations were found.

## Discussion

To our knowledge, this is the first study using a referential communication task to investigate two aspects of ToM ability (i.e., understanding others’ false beliefs and applying understanding of others’ false beliefs) in patients with schizophrenia. Our results show patients with non-remitted schizophrenia performed more poorly than controls in understanding another’s false belief, whereas no significant difference was found between remitted schizophrenia patients and controls. These findings are consistent with previous studies [18-22] in which remitted schizophrenia patients have exhibited intact ability to attribute others’ false beliefs. However, our results seem to contradict a study by Mo et al. [35], which reported that chronic stable schizophrenia patients performed worse than controls in a first-order false belief task. We speculate that this inconsistency may be the result of Mo et al.’s first-order false belief task involving more processing demands. In Mo et al.’s study [35], their first-order false belief task required participants to predict the characters’ behavior based on their false belief. According to the “response account,” reviewed earlier, succeeding in this type task requires not only the ability of false-belief-representation, but also the ability to execute the response-selection and response-inhibition processes [2]. In our task of understanding others’ false beliefs, the false belief probes were repeatedly presented to participants. Participants only provided a “yes” or “no” response to each probe. Thus, the simplicity of our task requirement might reduce the probability of participants responding incorrectly to false belief probes due to their prior knowledge of reality.

Our results show that patients with non-remitted schizophrenia and individuals with remitted schizophrenia performed significantly worse than controls on a task of applying knowledge of others’ false beliefs. While the experimental instruction had explicitly directed participants to take ZhangHong’s perspective during the task, patients with schizophrenia seemed to not consider the character’s belief about the contents in secretive-boxes. Our results are consistent with a previous study [25,36], which indicated that patients with schizophrenia had difficulties in taking others’ perspective. The findings are also consistent with previous studies using false belief picture-sequencing tasks [37-39]. Patients with schizophrenia performed poorly on this type of task that required the ability to apply others’ false beliefs. Based on these considerations, our results provide further evidence that schizophrenia is associated with a specific deficit in applying knowledge of others’ mental states. The deficit of applying others’ mental states may be an appropriate intervention target for schizophrenia during remission.

Our results also show no significant correlations between understanding/applying false beliefs and neurocognitive domain scores and clinical variables, including IQ, CPZ equivalents, Length of illness, VFT total score, and DST total score. We speculate that the lack of influence of these other variables may be due in part to the simplicity of our task. Moreover, we also added the rate of accuracy of false belief probe trials as a covariate into the ANCOVA for applying false beliefs. Thus, the difference in applying false beliefs between the two schizophrenia groups could not be completely accounted for by the inability to understand others’ false beliefs in non-remitted schizophrenia. Regarding the improvement in applying false beliefs found in remitted schizophrenia, we speculate this difference was due to a treatment effect from antipsychotic medication. Previous literature has reported that improvements on tasks involve applying false beliefs were observed when clozapine or olanzapine was administered [40].

Our results seem to support a dissociation of understanding others’ false beliefs from applying knowledge of others’ false belief in remitted schizophrenia. Patients with remitted schizophrenia showed deficits in applying others’ false beliefs, but appeared to retain normal ability in understanding others’ false beliefs. This conclusion might help to clarify confusion in the literature regarding whether ToM impairments in schizophrenia are trait-dependent or state-dependent. Evidence supporting a trait-dependent view of ToM impairments comes largely from studies in which false belief tasks required participants to use their knowledge of others’ false beliefs. For instance, studies by Langdon and Coltheart [41], Inoue et al. [23], and Anselmetti et al. [42] suggested that poor performance in schizophrenia on a false belief picture-sequencing task is attributable to stable, illness-related biological factors. On the contrary, the studies reporting a lack of association between ToM impairment and remitted schizophrenia primarily have used tasks that assess whether patients could understand others’ false belief [18-22]. Our results show that performances on a task of understanding others’ false belief was negatively associated with PANSS General symptoms, whereas no significant correlations were found between performance on a task of applying others’ false belief and general symptoms scores, positive symptoms scores or negative symptoms scores. These results might be explained by Frith’s proposal that schizophrenia is a “meta-representational disorder” that is only present in patients without remission [15], suggesting a state-related impairment. On the other hand, our results seem to indicate that defective ability to apply others’ mental states may be trait-dependent. However, the exact cognitive mechanisms underlying their disability of applying others’ false belief remain unclear. For example, we cannot exclude the possibility that difficulty in applying others’ false belief in schizophrenia was due to their limitation of generic executive processes, as proposed by Apperly et al. [10], which was used to explain the reason for normal adults’ failure in a similar task paradigm. Therefore, we preliminarily conclude that deficits in the ToM ability to attribute others’ mental states may be state-dependent, whereas deficits of the ToM ability to apply knowledge of others’ mental states may be trait-dependent.

The present study has two main limitations. First, no closely matched control tasks were adopted. Thus, no conclusions can be drawn regarding whether the patients’ poor performances on the task of applying others’ false belief are specific or of the result of general cognitive deficits in inhibiting interference. Secondly, in the present study, only the participant’s ability to understand others’ false belief and to apply this understanding were investigated, limiting our ability to compare our findings with results in previous studies using other types of mentalizsing tasks. However, in spite of these limitations, our findings may help to clarify findings in previous studies using false belief tasks.

## Conclusions

In summary, we investigated separately the ability to understand others’ false beliefs and the ability to apply understanding of the others’ false beliefs in both remitted schizophrenia and non-remitted schizophrenia with a revised computerized referential communication task. Our findings suggested a dissociation of understanding others’ false beliefs from applying others’ false beliefs in remitted schizophrenia. Though further work must be performed to determine the exact cognitive mechanisms underlying their disability of applying others’ false beliefs, present study might help to better understand the ToM deficits in schizophrenia. 

## Competing interest

Yong-guang Wang and other co-authors have no conflict of interest.

## Authors’ contributions

YGW and BHX participated in study design. YGW participated in study implementation. YGW and DLR wrote the paper. All authors read and approved the final manuscript.

## Pre-publication history

The pre-publication history for this paper can be accessed here:

http://www.biomedcentral.com/1471-244X/13/141/prepub
